# Supplementary material for: Are carbon emissions trading and green financial instruments synergistic? -Comprehensive quantitative research based on content analysis
Source: PLoS One. 2024 Mar 7;19(3):e0298601. doi: 10.1371/journal.pone.0298601 (PMC10919601; doi:10.1371/journal.pone.0298601)
Supplement: S1 Data — (ZIP) [file pone.0298601.s001.zip › Data/地级市Z_F工作报告.zip!/┴╔─■/╙¬┐┌╩╨/└┤╫╘╣1⁄2╓┌║┼í╛┬φ┐╦╩2╛▌═°í┐_2008.docx]

各位代表：

　　现在，我代表市人民政府向大会作政府工作报告，请予审议，并请政协委员和列席会议的同志提出意见。

　　一、过去五年工作的简要回顾

　　五年来，在省委、省政府和市委的正确领导下，在市人大、市政协的监督支持下，市政府团结带领全市人民，坚持以邓小平理论和“三个代表”重要思想为指导，全面贯彻落实科学发展观，奋发图强，锐意进取，圆满完成了市十三届人大一次会议确定的各项任务。2007年预计实现地区生产总值556亿元，比上年增长21.5%，五年年均增长18.4%。地方财政一般预算收入29亿元，增长41.6%，年均增长21%。城市人均可支配收入11858元，增长17%，年均增长12.9%。农民人均纯收入5750元，增长15%，年均增长11.7%。

　　（一）加快产业结构战略性调整，经济实力显著增强

　　农业和农村经济稳步发展。2007年完成农业增加值48.1亿元，增长8.3%，年均增长8.6%。粮食总产量72.2万吨，创五年新高；水产品产量42万吨，增长4.2%；肉、蛋、奶总产量31万吨，增长6.8%；设施蔬菜、水果面积发展到22万亩，“一村一品”专业村达到154个。农业产业化迈出新步伐。培育壮大龙头企业164户，农副产品加工率达到40%。农业标准化实现新突破。无公害、绿色、有机食品认证数量达到84个，比2002年增加70个。农业生产条件进一步改善。五年累计投入支农资金8.1亿元，改善灌溉面积60.9万亩，比2002年增加45.9万亩；植树造林54万亩，森林覆盖率达到43.3%；综合机械化水平达到51%。新农村建设扎实推进。60个试点村建设规划全部完成，一批惠民实事工程得到有效实施。县域经济实力明显提升。2007年各县（市）区地区生产总值增幅均在20%以上，2006年大石桥市被评为全国综合发展指数百强县（市）。

　　新型工业化进程明显加快。2007年全市新增规模以上工业企业186户，总数达到1200户，比2002年增加614户。实现规模工业增加值302.4亿元，增长28.5%，占地区生产总值的比重比2002年提高30个百分点。实现利税70亿元，增长30%，年均增长29.8%。六大产业集群实力显著提升，实现工业增加值232亿元，占工业总量的76.8%。科技创新能力不断增强，五年共安排500万元以上工业技改项目691项，是上个五年的2.3倍。建立市级以上企业研发中心64个，实施高新技术产业化项目184项，实现高新技术产业产值160亿元，增长60%，年均增长37.3%。累计开发新产品3777种，8种产品分别荣获中国名牌产品和全国驰名商标。大力发展循环经济，五年共实施资源节约与综合利用项目92项，单位地区生产总值能耗以年均3.8%速度递减，主要污染物排放控制在省计划以内。

　　第三产业呈现快速发展势头。2007年实现第三产业增加值186.5亿元，增长14.6%，年均增长13.4%。完成社会消费品零售总额143.4亿元，增长17%，年均增长15%。五年累计投资25.6亿元，新建各类市场51处，兴办农家（资）店895家。积极搞活商品流通，加强物价监控，市场秩序基本稳定。物流业快速发展，全市物流总量达到1.86亿吨，是2002年的3倍。信息、咨询、中介等新兴服务业不断壮大。旅游业发展步伐加快，2006年我市进入中国优秀旅游城市行列，2007年旅游总收入达到32.1亿元，增长82%，年均增长20.5%。金融安全区建设成效显著，本外币存款突破500亿元，年均增长15.5%；不良贷款比率降至13%，比2002年下降32.4个百分点；证券业得到规范，保险行业快速发展。2007年我市被评为全国金融生态城市。

　　项目开发建设取得重大成果。五年来，全市固定资产投资累计完成1193亿元，是上个五年的4.6倍，年均增长41.6%，其中工业固定资产投资743亿元，年均增长54.8%。新上投资1000万元以上项目1033项，其中亿元以上项目117项。鞍本集团鲅鱼圈新厂、中冶京诚（营口）中试基地、五矿中板宽厚板和华能电厂二期等一批重大项目相继开工建设，为全市经济持续快速发展奠定了坚实基础。

　　（二）全方位推进各项改革，经济社会发展活力不断增强

　　工业企业改革向纵深推进。坚持因企施策、有序推进的原则，加速实施国有企业转制和重组。五年共完成企业改制和盘活资产项目285项，分流安置职工6.7万人，盘活资产17亿元，基本完成国有企业产权制度改革任务。积极推进企业体制机制创新，加快建立现代企业制度，全市规模以上工业企业公司制改造面达到80%以上。大力发展民营经济，加速培育中小企业。2007年民营经济实现增加值420亿元，是2002年的3.1倍。

　　农村综合改革不断深化。认真落实惠农政策，共发放粮食直补和农机具补贴等项资金1.3亿元。进一步落实土地承包政策，引导土地经营权有序流转。全面推进集体林权制度改革，确权到户率、林权发证率超额完成省定标准。顺利完成农村税费改革，五年共免除税费9.3亿元。积极化解村级债务，农村债务由2002年的10.1亿元降至6.53亿元。

　　各项配套改革稳妥操作。深化行政管理体制改革，优化机构和编制设置，规范了行政审批程序。实施政府采购、国库集中支付等财政体制改革，公共财政服务能力进一步增强。搞好城市商业银行和农村信用社改革，资产质量和管理水平不断提高。

　　（三）大力推进沿海经济带开发建设，对外开放实现新突破

　　沿海经济带开发成效显著。以“一港、三区、两基地”为支撑，整体推进1600平方公里沿海经济带建设。大力实施以港兴市战略，五年累计完成投资110亿元，完善了港口功能及设施。2007年实现货物吞吐量1.2亿吨，集装箱运量135万标箱，分别是2002年的3.8倍和4.5倍，营口港已跻身全国沿海十大亿吨港口行列。沿海产业基地迅速崛起，一期44平方公里的基础设施基本建成，富士康营口科技园等40个重点项目相继开工建设，完成投资87亿元。开发区经济实力显著提升，规模以上企业达到203户，注册外资企业达到450户，2007年地区生产总值实现175.5亿元，比上年增长30%，年均增长40.5%。高新区技工贸总收入实现67.2亿元，增长50%，年均增长72%。仙人岛能源化工区起步区基本实现“六通一平”，大石桥已成为全国乃至世界镁质耐火材料研发、生产、销售基地。

　　外向型经济实现新跨越。五年来，全市共引进外商投资项目684个，其中500万美元以上项目65个，1000万美元以上项目31个；实际直接利用外资7.3亿美元，年均增长30.4%（可比口径）。引进内联资金469.7亿元，实施500万元以上内联项目1180个。2007年完成外贸进出口总额18.9亿美元，其中出口13.5亿美元，增长15%，年均增长22.2%。

　　（四）全面推进和谐营口建设，民生工程和社会事业取得新成就

　　民生工程建设卓有成效。五年来，市政府共筹集资金45.9亿元，有效实施了惠及城乡群众的利民举措，一些多年积累的民生问题得到较好解决。积极落实各项扶持政策，实现就业和再就业36.7万人次，转移农村剩余劳动力13.4万人次，城镇登记失业率由5.9%下降到3.3%。养老保障体系日趋完善。累计支付养老金44.6亿元，参加养老保险人数达到37.8万人。五年共发放低保金4.3亿元，享受低保人数达到13.6万人，实现了应保尽保。107万农民纳入新型农村合作医疗，参合率达到90%。城镇职工基本医疗保险参保人数达到46.5万人，56681名国有集体困难企业退休人员进入保障范围。建立了农民工工资保证金制度，切实维护了农民工合法权益。覆盖城乡的救助体系基本形成，五年共筹集款物折合资金1.05亿元，救助困难群众61.5万人次。发放小额担保贷款6207万元，社保等各类补贴近3亿元，极大地改善了人民群众的基本生产生活条件。

　　各项社会事业健康协调发展。五年共投入财政性教育资金26.8亿元，改造中小学危房121所，新建校舍63.5万平方米。发放就学资助资金1329万元，惠及45万名贫困学生。五个县（市）区通过了“双高普九”验收，新一轮“普九”工作走在全省前列。2007年全市普通高中和职业院校在校生分别达到4.7万人和3.6万人，是2002年的2.2倍和1.8倍。累计投入财政资金7.3亿元，新建了市中心医院和传染病院，改扩建城市社区卫生服务机构和乡镇卫生院114所，有计划整合了卫生资源，促进了公共卫生和动物疫情防控体系建设，有效控制了非典、禽流感等重大疫情。五年累计投入资金2.3亿元，改善了城乡文化基础设施，促进了文化事业的健康发展。体育健身设施走进社区，全民健身活动广泛普及，竞技体育取得显著成绩。人口计生服务能力进一步增强，出生人口结构趋于合理，人口出生率控制在5.52％。以内。广播电视网络化进程加快，全市91%的村开通了有线电视。精神文明创建活动扎实开展，和谐社区建设成果显著，全民文明素质不断提高，2007年我市进入省文明城市行列。双拥工作成果喜人，我市连续两次获得全国双拥模范城称号。国防建设不断加强，在全国首创国防教育基金和教育基地，全民国防意识进一步强化。民兵预备役、国家安全、民族宗教、外事侨务、档案、妇女儿童、红十字、老龄和残疾人等项工作得到进一步加强。

　　（五）城乡面貌明显改观，发展环境进一步改善

　　城市建设和管理水平不断提高。五年来，全市基础设施建设总投资122.8亿元，是上个五年的5.3倍。出让土地使用权和采矿权621宗，获土地出让金和价款23.4亿元。实施棚户区改造、廉租房建设115万平方米，完成房地产开发面积1100万平方米，城镇人均住房面积由14.7平方米提高到24.5平方米。改造旧住宅小区49个，受益居民5万余户。滨海热电厂、污水处理厂等一批公益性项目投入使用，鲅鱼圈水源和供水管网一、二期改造等工程全部完工，新增日供水能力12.6万立方米。新建和改扩建青花大街、金牛山大街等城市道路186条，道路完好率由2002年的43%提高到90%，大公交网络基本形成。完成滨海路、营柳路等公路建设和改造2487公里，公路密度比2002年提高14个百分点。全市发电装机容量达到201万千瓦，营口电网开始由220千伏向500千伏等级的升级。加大城市综合整治力度，市容市貌、环境卫生、市场管理、交通秩序明显改观，城市美化、绿化、亮化、净化水平不断提高，物业管理逐步走向规范化。市区空气、声音环境质量居全省前列，城市污水处理达到国家二级排放标准。

　　投资环境得到明显改善。五年来，相继开展了“项目年”、“招商引资年”、“科学发展年”、“工业发展年”等主题活动，出台了一系列促进软环境建设的政策规定。大力削减行政审批事项，市级审批项目由五年前的1573项减少到466项。广泛实行一条龙服务和一站式办公，提高了机关办事效率和服务水平，营造了有利于投资创业的良好环境，我市进入全国投资环境百佳城市行列。

　　（六）切实加强民主和法制建设，政府科学执政、依法行政水平不断提高

　　民主法制建设进一步加强。自觉接受人大依法监督和政协民主监督，充分发挥各民主党派、工商联、无党派人士和人民团体的参政议政作用，广泛听取社会各界人士的意见和建议。五年共办理人大代表建议、议案和政协委员提案2211件，办复率100%，满意率96.7%，办结率76.2%。深入开展“四五”、“五五”普法活动，依法治市进程不断加快，全民法律素质不断提高。全面开展“平安营口”创建活动，严厉打击各种犯罪活动，刑事、治安案件发案率呈逐年下降趋势。继续实施领导包案责任制，搞好矛盾纠纷调处，全地区群众来信来访量下降44.8%。加强隐患排查和应急处理工作，安全生产保持平稳态势。

　　依法行政和科学执政水平不断提高。认真落实《全面推进依法行政实施纲要》，加快法治政府建设。严格按照法定权限和程序履行政府职责，从制度上保证行政行为的公开、公平、公正。创新政府管理体制，强化行政效能监察，社会管理和服务职能不断强化。政府班子及成员自觉遵守廉洁自律的各项规定，树立了廉洁高效的政府形象。

　　上述成绩的取得是市委正确领导的结果，是市人大依法监督、市政协民主监督的结果，是各民主党派、工商联、无党派人士、人民团体、中省直单位、驻营部队、武警官兵关心、帮助的结果，是全市人民发奋图强、共同努力的结果。在此，我代表市政府向全市广大干部群众，向所有关心支持营口建设的同志们、朋友们表示崇高的敬意和衷心的感谢！

　　各位代表，本届政府任期的五年，正值东北老工业基地振兴战略和辽宁“五点一线”开发战略全面实施的重要阶段。五年来，我们牢牢把握东北振兴和沿海开放的双重机遇，创造性的提出并全力实施了沿海经济带开发战略，有效破解了长期制约营口发展的一系列重点问题，实现了港口建设与发展的新跨越；实现了以产业基地为代表的沿海经济带建设的重大进展；实现了以鞍本集团鲅鱼圈新厂为重点的大项目建设的重大突破；实现了传统优势产业与新兴重化工业的整体推进；实现了国民经济由恢复性增长转入持续快速健康发展。实践证明，过去五年是我市优化经济结构取得突破性进展的五年，是改革开放进一步深化的五年，是城乡综合实力显著增强、社会更加和谐的五年，是夯实基础、增强发展后劲的五年，也是人民群众得到实惠最多的五年。

　　回顾五年的发展历程，市政府工作主要把握了以下几点：一是始终坚持以中国特色社会主义理论为指导，用科学发展观统领经济社会发展全局，努力实现城乡建设同步推进，经济社会协调发展；二是始终坚持不断解放思想，以实事求是和与时俱进的精神推进思维创新，用改革开放的方法着力解决发展中的关键问题；三是始终坚持以港兴市和工业强市战略，举全市之力支持港口做大做强，集中精力抓好项目开发和产业培育，千方百计积蓄经济发展后续力量；四是始终坚持以人为本，高度关注并认真解决群众最关心、最迫切、最现实的生产生活问题，让改革发展成果惠及最广大的人民群众；五是始终坚持强化政风建设，促进政府组成人员和广大机关干部保持昂扬向上的工作热情和高度负责的敬业精神，求真务实，埋头苦干，不事张扬，形成了推动营口全面振兴的强大合力。

　　在肯定成绩的同时，我们也清醒地看到，全市经济和社会发展中依然存在一些亟待解决的困难和问题：一是农业产业化、市场化程度较低，农民持续增收的渠道不宽。二是工业企业科技创新能力不强，节能减排压力较大。三是对外开放整体水平不高，利用外资和出口创汇规模较小。四是财政收入总量偏小，刚性支出不断增加，收支矛盾仍很突出。五是资源保护、环境治理需要进一步加强，生态文明建设的任务十分艰巨。六是民生工程实施仍面临诸多问题，城乡部分群众生活还很困难，和谐社会建设任务相当繁重。七是人才匮乏对经济社会发展的制约日见突出，劳动力资源结构性短缺已开始显现。八是政府自身建设还不适应新形势、新任务的要求，发展环境有待进一步改善。对此，在今后的工作中，我们将采取有效措施，认真加以解决。

　　二、今后五年和2008年工作的主要任务

　　未来五年，是我市深入贯彻落实党的十七大精神，全面建设小康社会的关键时期，也是实现营口老工业基地全面振兴的决定性阶段。站在新的历史起点上，我们必须抓住机遇，乘势而上，以更大的魄力推进各项工作，圆满完成“十一五”规划确定目标，实现“十二五”高起点开局，努力开创我市改革开放和现代化建设的新局面。

　　今后五年政府工作的总体思路是：全面贯彻落实党的十七大精神，以邓小平理论和“三个代表”重要思想为指导，以科学发展观为统领，按照市第十次党代会的总体部署，继续实施以港兴市、工业强市战略，突出抓好加快新型工业化进程、推进社会主义新农村建设和构建和谐营口三大重点任务，深化改革开放，推进科技创新，优化产业结构，改善城乡环境，着力促进经济发展方式转变；坚持以人为本，高度关注民生，切实改善人民群众生活质量和水平，努力提高社会文明程度，推动经济社会全面协调可持续发展，为建设沿海经济强市奠定坚实基础。

　　今后五年工作的奋斗目标是：

　　——国民经济实现又好又快发展。到2012年，地区生产总值年均增长18%以上；单位地区生产总值能耗年均递减4%；地方财政一般预算收入年均增长21%。综合经济实力大幅提升，经济发展的协调性和可持续性进一步增强。

　　——沿海经济带建设实现新突破。到2012年，沿海产业基地一期44平方公里的开发初具规模，现代化滨海新区框架基本形成，地区生产总值达到300亿元，初步建立具有较强竞争力的产业体系。开发区地区生产总值力争达到640亿元，年均增长30%。高新区、仙人岛能源化工区、船舶工业园等各类园区和镁质材料基地成为我市未来重要的经济增长极。

　　——对外开放质量和水平大幅度提升。到2012年，港口综合竞争力进一步增强，港口吞吐量突破2.5亿吨，集装箱运量300万标箱。全市实际直接利用外资年均增长30%；外贸出口年均增长15%。努力拓展对外开放广度和深度，参与国际经济合作和竞争新优势基本形成。

　　——产业结构调整取得重大进展。到2012年，现代工业体系框架基本形成，六大产业集群更加完善，成为国内重要的精品钢材、能源化工、装备制造、船舶修造及配套产业基地，规模工业增加值达到1000亿元。传统服务业发展水平显著提升，现代服务业迅速壮大，社会服务功能不断增强。

　　——社会主义新农村建设进程加快。以农业标准化、市场化和产业化为重点，大力发展现代农业，农产品加工率达到48%，农业增加值年均增长10%。强化农村基础设施建设，加快城镇化进程，繁荣壮大县域经济，构建城乡经济社会一体化发展新格局。

　　——城乡环境显著改善。进一步调整城乡规划布局，拓展城市发展空间，增强城市综合承载能力，到2012年城市化率达到65%。着力推进资源节约型、环境友好型社会建设，创造优美舒适的城乡人居环境，实现人与自然和谐发展。

　　——和谐营口建设取得更大成效。按照“学有所教、劳有所得、病有所医、老有所养、住有所居”的总体要求，建立起覆盖城乡居民的民生保障体系，社区服务水平全面提升，社会管理机制更加完善，和谐稳定的社会环境进一步形成。

　　——各项社会事业协调发展。教育、文化、卫生、体育等事业取得明显进步，基本形成较为完善的现代国民教育体系、科技和文化创新体系、全民健身和医疗卫生体系，公共服务能力显著增强。

　　——人民生活水平逐年提高。到2012年，城市人均可支配收入达到20900元，年均增长12%；农民人均纯收入达到9260元，年均增长10%。人民富裕程度普遍提高，人居条件和生活质量明显改善。

　　2008年是新一届政府的届首之年，做好今年工作对完成今后五年任务具有十分重要的意义。我们要认真贯彻中央经济工作会议和市委十届六次全会精神，把握大局，突出重点，确保实现经济社会又好又快发展。2008年全市主要奋斗目标是：地区生产总值667亿元，增长20%；固定资产投资483亿元，增长15%；实际直接利用外资1.72亿美元，增长30%；外贸出口15.5亿美元，增长15%；地方财政一般预算收入36.3亿元，增长25%；城市人均可支配收入13280元，增长12%；农民人均纯收入6440元，增长12%；城镇登记失业率控制在3%以内。

　　围绕实现上述目标，今年要重点抓好以下九项工作：

　　（一）强力推进沿海经济带建设，加速构建开放型经济新格局

　　全面提升港口牵动和辐射功能。继续实施以港兴市战略，不断加大对港口发展的支持力度，推进仙人岛港区、疏港铁路、公路和出海产业大道建设，确保四期工程和A港池2个成品油泊位年底投入使用。加快构建覆盖东北腹地的物流网络体系，提高港口的管理与服务水平。全年完成货物吞吐量1.4亿吨，增长16.7%；集装箱160万标箱，增长18.5%。

　　全力推进沿海产业基地建设。以打造集约型、节约型、生态型滨海新城区为目标，加快奥体中心、商务大厦、文化艺术中心等基础设施建设，完善市政及配套设施，搞好区内绿化、美化，努力营造项目入驻和人口集聚的良好环境。强化外引内联工作，提高项目签约率、开工率和投产率，全年力争新开工项目50个，完成固定资产投资80亿元，确保中冶京诚和大族冠华等一批重点项目投产运行。

　　努力实现开发区经济的跨越式发展。开发区要围绕提升城市品位，积极发展现代服务业，进一步完善城市功能，全力推进红海新区建设。坚持以大高外项目、环保项目和高附加值项目引进为重点，努力提高招商引资水平。全年确保开发亿元以上项目30个，主要经济指标增长30%以上，逐步成为全市对外开放的先导区、新型工业化项目的集聚区和临港经济的示范区。

　　促进重点区域快速开发。高新区要在逐步完善船舶制造等五大产业体系的基础上，突出抓好企业技术中心和公共技术平台建设，加快区域高新技术产业集群成长，全年主要经济指标增长50%以上。仙人岛能源化工区要坚持基础建设与项目引进同步推进，全年完成基础设施投入2.8亿元，确保3平方公里起步区具备项目入驻条件。积极推进大石桥有色金属工业园开发建设，尽快形成较为完善的产业体系。

　　大力发展开放型经济。建立和完善以企业为主体、专业队伍为骨干、中介组织为辅助、政府组织协调的招商机制。实施“走出去”战略，组织策划好大型招商、项目推介和展洽活动。全年力争签约和开工建设500万美元以上项目19项，其中1000万美元以上的大项目10项。坚持以质取胜的方针，进一步优化出口商品结构，努力扩大机电产品、高新技术产品、农产品和具有自主知识产权的产品出口，增强开拓国际市场的能力。

　　（二）继续实施工业强市战略，进一步提升工业经济整体实力

　　强化项目支撑作用。围绕做大做强支柱产业、加快发展骨干企业和优化产品结构，着力扩充项目储备，积极推进外引内联，促进一批重大项目的实施。抓紧筹划中小项目园区，拓展中小项目发展空间。全年确保实现内联引资220亿元，开工建设投资1000万元以上工业项目200个，新增规模企业160户，工业增加值增长30%。

　　培育壮大产业集群。以六大产业为基础，推动产业配套和项目延伸，进一步拉长产业链条。加快鞍本集团鲅鱼圈新厂和五矿宽厚板项目建设，带动精品钢材配套及深加工项目引进；推进与中海油、中石油等大企业合作，促进精细化工系列产品开发；突出抓好中冶京诚等大型装备制造、辽宁船舶工业园和沙岗船舶修造及配套等重点行业发展。同时，在提升镁质材料产业结构、增强纺织服装业创新能力、提高新型建材业发展水平方面努力实现新突破。全年六大产业集群总产值达到1050亿元。

　　加速实现企业技术创新。围绕增强企业创新能力，加大政策扶持力度，引导企业加大投入。全年实施500万元以上技改项目120项，完成投资65亿元。加快镁质材料、纺织服装两个共用技术平台开发，积极组建企业研发中心，全年新建市级以上企业研发中心20个；组织实施技术创新项目100项；新增高新技术企业30家，高新技术产业增加值达到54亿元。继续实施名牌战略，年内新创省级以上名牌9个，省级以上著名商标10件。

　　大力推进节能减排。以建设省级循环经济试点市为契机，加强重点行业节能降耗工作，全年实施工业资源节约与综合利用项目25项。建立节能减排指标考核体系，严把项目能效、环评关口，坚决淘汰“三高型”落后生产能力。单位地区生产总值综合能耗下降4.5%以上，主要污染物化学需氧量、二氧化硫排放量分别减少3.2%和2.4%。

　　继续深化企业改革。抓住重点、难点企业，扎实做好国有企业改革收尾工作。加强国有资产监管，提高国有资本运营效益。引导民营企业加速建立现代企业制度，着力解决产权不清等历史遗留问题。完善中小企业服务体系和创业成长机制，并在国债项目、技改贴息、信用担保等方面予以支持。

　　（三）发展壮大县域经济，扎实推进新农村建设

　　促进县域经济快速健康发展。突出发展县域工业，积极引导资金、技术、人才等要素向小城镇和园区集聚，加快县域工业向规模化、集约化方向发展，实现工业反哺农业。在着力推进中心镇建设的同时，有重点地培育一批具有产业特色的中小城镇，不断提升农村城镇化水平。用好用足省县域经济重点县、产业项目贴息等优惠政策，确保县域经济总量和效益的增幅超过全市平均水平。

　　大力发展现代农业。不断调整优化农业结构，积极发展设施农业、订单农业和创汇农业。全年粮食播种面积保持在146万亩，蔬菜、水果、水产品实现稳定增长，畜牧业产值占农业总产值比重达到30%。大力推进农业产业化经营，扶持龙头企业发展壮大，全年新建、续建500万元以上项目20个。加快农业标准化建设，无公害、绿色、有机食品认定面积达到128万亩。抓紧做好优质农产品商标注册工作，力争有更多的品牌载入国家和省农业名牌产品目录。

　　加大新农村建设投入。采取财政和社会支持等多种方式，努力增加农业投入。以农业基础设施建设为重点，着力改善农村生产生活环境。搞好河道整治和水库除险加固，全年新增灌溉面积1.8万亩，治理水土流失面积8万亩。大力发展生态经济型林业，完成植树造林3.6万亩。优先发展保护性耕作机械，综合机械化水平提高0.5个百分点。加大水资源、基本农田、海岸线和海洋渔业资源保护力度，认真解决农村饮用水安全问题，推进农村清洁能源建设。加强气象预报预警工作，切实做好防灾减灾。积极推进试点村建设，充分发挥示范、辐射作用，提高新农村建设水平。以东部山区为重点，认真抓好扶贫开发工作，推动农村剩余劳动力多渠道转移，促进山区经济社会快速发展，改善群众生产生活条件。

　　稳妥推进农村改革。巩固新一轮农村土地延包成果，探索建立多种形式的土地流转机制。抓好村级不良债务清理试点工作，有效化解村级债务。加大监督检查力度，实行村务公开，切实减轻农民负担。继续深化农村综合配套改革，增强农村经济社会发展活力。加快发展各类专业合作经济组织，全年新建农业专业合作社100个。完善农业科技推广体系，加大科普和培训工作力度，积极培育农村实用人才。

　　（四）大力发展现代服务业，强化城市综合服务功能

　　努力构建现代服务业体系。进一步完善服务业发展规划，继续推进“万村千乡工程”，优先发展社区商业，吸引国内外知名商家进驻营口。开发连锁、配送等现代经营形式，规范中介服务机构，引入信息、咨询、会展等新型业态。完善港口保税物流中心、沿海产业物流基地、交通物流中心网络体系，推进商贸物流网络与辽宁中部城市群物流网络对接。

　　加快旅游资源整合与开发。充分利用山、海、林、泉、寺等特色资源，开辟温泉度假、乡村旅游和海河风光等特色项目，全力打造“百年商埠、河海之滨、敬母圣地”的旅游品牌，提升管理和服务水平，努力构建起布局合理、特色突出、开发有序、配套完善的旅游发展新格局。

　　（五）高度关注民生民情，推进和谐营口建设

　　积极构建覆盖城乡的社会保障体系。以创建充分就业城市为目标，拓宽就业渠道，强化就业培训，全年实现实名制就业6万人。严格执行《劳动合同法》，建立外来务工人员的保障机制，努力改善用工环境，解决劳动力结构性供需矛盾。继续完善城乡养老保障机制，全年养老保险扩面5万人。建立健全以化解城乡群众大病风险为重点的医疗保障体系，城镇职工基本医疗保险扩面5万人。认真落实住房保障制度，建立健全解决城乡贫困家庭住房难问题的长效机制。完善最低生活保障制度，加强社会救助体系建设，做到低保对象应保尽保，各类困难群众得到及时救助。切实强化市场价格监管，千方百计保证副食品供应，严厉打击哄抬物价等违法行为，确保价格总体水平基本稳定，安排好人民群众生活。

　　继续推进法治营口建设。深入开展“五五”普法教育和依法治市工作，增强全社会法律意识，形成依法决策、依法行政、依法管理经济和社会事务的良好环境。全面开展平安创建活动，加强社会治安综合治理，建立预防、管理和打击的综合体系，为经济建设和社会发展创造良好的治安环境。

　　努力维护社会和谐稳定。充分发挥信访、市民投诉中心和民心网的职能作用，畅通群众诉求渠道，严格落实信访工作责任制，有效化解社会矛盾。进一步拓展社区职能，充分发挥街道、居委会促进和谐营口建设的积极作用。高度重视安全生产和公共安全，及时消除各种隐患，遏制重特大事故发生。建立反应迅速的应急预警机制，妥善处理各类突发事件。

　　（六）加强城乡环境建设，改善和优化人居环境

　　进一步加强城乡规划和管理。科学搞好《城市总体规划》新一轮修编，完成各类工业园区和城市重点区片的控详规划及100个村庄规划任务。认真贯彻《规划法》，规范规划管理行为，坚决维护规划的权威性和严肃性。加大综合执法力度，全面治理各种与城市发展不相协调的行为。大力推进物业管理四级网络建设，促进管理重心下移，实现小区物业管理全覆盖。加强城市生态建设，以主次干道两侧和小区绿化为重点，大力实施城市绿化工程。积极推进节能光源建设，提高城市亮化水平。坚持“控制总量、盘活存量、提高质量”的原则，继续完善土地招、拍、挂出让制度，规范房地产开发和交易秩序。以争创国家环保模范城为目标，进一步加强环境保护，严格实施环境评价和“三同时”制度，搞好污水、噪声、烟尘和废气的综合治理，确保饮用水源水质优良，环境噪声功能区达标率95%，市区空气质量达到二级标准。

　　集中力量为群众办好实事。以解决关系群众切身利益问题和促进社会事业发展为重点，努力办好“十件实事”：1、改造农村中小学校舍21所；2、改扩建农村乡镇卫生院19所；3、培训农村实用技术人才1000人；4、新建乡镇消防站8座；5、建设经济适用住房40万平方米、廉租房3万平方米，改造旧小区10个；6、改造市区街巷道路30条；7、新建营口市光荣院、流浪未成年人救助保护中心，改造老年人活动中心；8、新建营口市博物馆；9、改扩建庄林线营口至盖州段、沈营线营口至高坎段和建设营柳路至庄林线的营滨路；10、实施焦化燃气综合利用，新增煤气用户1万户。建设“五大工程”：1、奥体中心建设工程；2、西炮台公园扩建工程；3、渡口老街改造工程；4、城市污水处理二期工程；5、大伙房水库输水配套工程。同时，进一步加强能源基础设施建设，加快推进西部热电厂二期、石门水库等续建工程。

　　（七）认真做好财税金融工作，保障资金有效供给

　　进一步加强财源建设。继续完善激励机制，综合运用贷款贴息、财政补助、信用担保等现行政策，支持中小企业发展和重点项目建设，培植基础财源和新兴财源。规范税收征管，加强对重点企业和重点税种的有效监控，加大国有资产有偿使用收入、国有资本经营收益、特许经营收入等征收力度，做到应收尽收。

　　不断优化财政支出结构。重点向“三农”、社会保障、公共教育、基础科研事业、循环经济和促进区域经济协调发展倾斜，构建以公共化为取向、以均等化为目标、以规范化为原则的公共财政体制。继续压缩消费性支出，节约公用经费。规范政府举债、担保行为，建立债务预警和偿债机制，规避财政风险。

　　努力提高金融业竞争力。进一步改善金融生态环境，加强信用体系建设，防范新的金融风险。引导金融机构不断深化改革，积极开展金融创新，提高服务水平。加强和完善担保体系，认真做好企业上市工作，拓宽企业直接融资渠道，吸引外埠银行在我市设立机构。支持保险业规范健康发展，开展农业保险试点。

　　（八）大力发展各项社会事业，实现经济社会协调发展

　　优先发展教育事业。以提高素质教育水平为重点，合理配置教育资源，整体推进新一轮“普九”工作，巩固高中阶段教育成果，重视学前教育、民族教育，关心特殊教育，规范民办教育，提高高等教育办学层次和水平，促进教育公平和均衡发展。大力发展职业教育，努力打造具有营口特色的示范性职业教育体系。完善城乡教育经费保障机制，全面落实“两免一补”政策。加强人才引进和智力开发，开通人才引进的绿色通道，优化人才队伍结构。

　　加快发展文化、卫生、体育等项社会事业。以构建公共文化服务体系为重点，不断丰富城乡群众文化生活，大力发展文化事业和文化产业。加强文化市场监管和文物保护，繁荣、净化文化事业发展环境。深化医疗机构内部改革，加强社区医疗卫生服务，规范新型合作医疗管理，确保城乡群众病有所医。高度重视重大疫情防控和食品药品安全，努力提高全民健康水平。改善人口结构，提高人口素质，稳定低生育水平，人口出生率控制在6.11％。以下。深入开展全民健身活动，不断提高竞技体育水平。全面完成有线电视村村通工程，推进数字电视整体转换试点工作。深入贯彻《全民科学素质行动计划纲要》，广泛搞好科学技术普及。认真落实民族宗教政策，促进民族宗教事业发展。做好妇女儿童、老龄、残疾人、红十字、外事、侨台、档案等项工作。

　　深入开展精神文明创建活动。积极倡导“诚信、和谐、务实、创新”的营口精神，弘扬社会正气，培育文明新风，全面提升市民文明素质和城乡文明程度。以强化海防为重点，进一步加强国防建设，增强全民国防观念。认真做好国家安全和民兵、预备役及人防工作，不断提高双拥共建水平。

　　（九）进一步提高执政能力，建设人民满意政府

　　加强民主法制建设。自觉接受人大依法监督和工作监督，认真执行和落实人大常委会的各项决议和决定。加强与政协的联系和沟通，积极支持政协履行政治协商、民主监督、参政议政职能。提高人大代表建议和政协委员提案办理质量。增进与民主党派、工商联、无党派人士和人民团体的联系。大力推行政务公开，保障公民的知情权、参与权和监督权。强化依法行政意识，全面提高行政执法水平。

　　加强服务环境建设。以行政审批服务中心运行为契机，深入开展“环境建设年”活动，着力解决影响和制约发展的突出问题，建立和完善县（市）区行政审批便民服务网络体系，全面推行一门受理、联合审批和限时办结制度，努力打造优质、高效、便捷、规范的服务平台，为全市经济社会发展创造宽松的服务环境。

　　加强政府执行力建设。坚持把维护群众利益摆到突出位置，加速转变政府职能，把更多精力用在促进社会发展和改善民生上来。进一步增强大局意识，防止和克服本位主义，确保政令畅通。建立健全机关绩效考核机制，促进责任政府建设，狠抓各项工作的落实。不断加强作风建设，坚决反对形式主义、官僚主义，不搞劳民伤财的形象工程，不做华而不实的表面文章，以良好作风影响和带动群众，提高政府的诚信度和公信力。

　　加强廉政建设。坚持标本兼治、综合治理、惩防并举、注重预防的方针，强化源头防范，加大对违法违纪案件的查办力度，切实解决重点行业、重点领域损害群众利益的突出问题。发扬艰苦创业、勤俭办事的传统，坚决纠正奢侈腐败、铺张浪费之风。加大监察、审计等专门机关的监督力度，强化对行政权力运行的制约和监督，建设廉洁政府。

　　各位代表，回顾过去，营口老工业基地全面振兴已打下坚实基础；展望未来，全面建设小康社会的神圣使命无上光荣。让我们在党的十七大精神指引下，在市委的正确领导下，团结和带领全市各族人民，万众一心，开拓奋进，为建设人民富裕安康、社会安定和谐的沿海经济强市，开创营口的美好未来而努力奋斗！
